# Cross-Sectional Survey on Quitting Attempts among Adolescent Smokers in Dharan, Eastern Nepal

**DOI:** 10.1155/2016/6859291

**Published:** 2016-09-22

**Authors:** Pranil Man Singh Pradhan, Kedar Marahatta

**Affiliations:** ^1^Department of Community Medicine and Public Health, Maharajgunj Medical Campus, Institute of Medicine, Tribhuvan University, Kathmandu, Nepal; ^2^World Health Organization (WHO) Country Office, Kathmandu, Nepal

## Abstract

*Background*. Adolescents frequently attempt smoking cessation but are unable to maintain long term abstinence because they are dependent on nicotine and experience withdrawal symptoms.* Objectives*. This study aimed to explore the quitting attempts among adolescent smokers in Dharan Municipality of Eastern Nepal.* Methods*. A cross-sectional study was conducted using pretested self-administered questionnaire adapted from Global Youth Tobacco Survey to assess current smokers and quitting attempts among 1312 adolescent students in middle (14-15 years) and late adolescence (16–19 years). Chi square test was used for association of various factors with quitting attempts.* Results*. The prevalence of current smoking was 13.7%. Among the current smokers, 66.5% had attempted to quit in the past because they believed smoking was harmful to health (35.5%). The median duration of quitting was 150 days. Nearly 8% of the current smokers were unwilling to quit in the future because they thought it is already a habit (60%). Smokers who are willing to quit smoking in the future were more likely to have made quitting attempts (OR = 1.36, 95% CI = 0.40–4.45).* Conclusion*. Relapse often occurs even after multiple quitting attempts. Tobacco focused interventions to support abstinence are important during adolescence to prevent habituation.

## 1. Introduction

Continuum of smoking behavior among children and adolescents can be described in stages of preparation, trying, experimentation, regular smoking, and nicotine dependence [1]. Adolescent smokers often want to stop smoking and frequently attempt smoking cessation but are unable to maintain long term abstinence mainly because they are dependent on nicotine and experience withdrawal symptoms just like adults [2]. Continuous use of a substance despite knowing its harm is not because of person's weakness or lack of willpower but has a biological basis of dependence [3]. Nicotine dependence is characterized by tolerance for nicotine and strong craving and desire to smoke along with withdrawal symptoms if an attempt is made to quit. So there is a high probability of relapse after quitting [1]. Nicotine is typically the first substance of abuse youths encounter, and adolescents who use tobacco are 15 times more likely to progress to alcohol and other drug use than those who abstain from tobacco [4].

Tobacco dependence is known to be a chronic condition and only few of those who initiate quitting become successful. DiClemente et al. proposed the stages of change which represent the temporal, motivational, and constancy aspects of change that the smokers cycle through. These four stages are precontemplation, contemplation, action, and maintenance [5]. According to Knight, the stage of preparation exists before the action stage [6]. In the stage of precontemplation, smokers are not planning to quit smoking in the short term future. In the contemplation stage, smokers have progressed to thinking about quitting. The preparation stage consists of smokers who plan to quit smoking. The action stage includes smokers who have quit smoking or who are in the process of quitting and the maintenance stage represents smokers who have remained abstinent [4]. With relapse, the stages of action or maintenance are broken and the smoker begins the cycle from precontemplation. Although the adolescent smokers travel through these stages in the same way as adults, their ability to quit smoking is largely determined by the nicotine dependence and withdrawal symptoms that they experience [4]. This study aimed to explore the proportion of current adolescent smokers with unsuccessful quitting attempts and various factors associated with the quitting attempts.

## 2. Methods

### 2.1. Study Setting and Data Collection

This cross-sectional study was conducted in Dharan Municipality of Sunsari district of Nepal [7]. From the list of 87 schools of Dharan (80 private schools and 7 government schools), stratified random sampling was carried out with proportionate allocation according to the type of schools. Fifteen private and two government schools were randomly selected based on population proportionate to size with an assumption of 100 students per school. This was followed by random selection of classes from the selected schools and all the students of the selected class were included in the study. The final sample of the study was 1312 adolescent students considering full participation and submission of completed questionnaires.

Data collection for this study was carried out using pretested, self-administered questionnaire adapted from Global Youth Tobacco Survey (GYTS). The questionnaire was designed after necessary modifications in order to make it more suitable for local setting. Briefing was done prior to administration of the questionnaire to make it more understandable for the students.

### 2.2. Outcome Measures

Outcome measures for this study were current smoking and quitting attempts. Current smokers were identified as students who smoked cigarettes on one or more days of the 30 days preceding the survey. Quitting attempt was determined from the question, “Have you ever tried to quit smoking cigarettes in the past?” Students who gave affirmative response to this question were considered to have made quitting attempt.

### 2.3. Data Management and Analysis

Collected questionnaires were thoroughly checked by the researchers to avoid inconsistencies. Data was entered into Microsoft Excel in the form of codes and analyzed using Statistical Package for Social Sciences (SPSS Version 17) (SPSS Inc., Chicago, Illinois, USA). Descriptive statistics was used to analyze the outcome variables. Chi square test was used to explore the association of various independent variables with quit attempts. Level of significance was set at 5%.

### 2.4. Ethical Consideration

The ethical approval for this study was obtained from the Institution Ethical Review Board of B.P. Koirala Institution of Health Sciences, Dharan, Nepal. Informed consent was obtained from each participant and written permission was taken from the school authorities before interaction with the students. Participation in the study was voluntary and full confidentiality of the responses was maintained. The researchers ensured absence of any school personnel during the process of collection of data in order to avoid response bias.

## 3. Results

The prevalence of current smokers was 13.7% (182/1312). Among the 182 current smokers, 66.5% had attempted to quit smoking in the past. Majority of the current smokers had attempted to quit in the past because they believed smoking was injurious to health (35.5%) followed by instruction from the parents to quit (21.5%) and did not want to continue smoking (10.7%). Other reasons behind quitting attempts were not having enough time to smoke, advice from the doctor to quit smoking, and residing in hostel (Table 1).

The median duration of quitting smoking was 150 days (interquartile range 60–315). The duration of quitting smoking ranged from minimum of 3 days to maximum of 5 years.

Majority of the current smokers expressed the willingness to quit smoking in the future (91.8%). Among those students who were not willing to give up, the reasons were explored. The majority of them (60%) were unwilling to give up thinking that smoking is already a habit. One fifth of them thought they cannot succeed in giving up smoking and nearly 13% thought that they cannot live without smoking (Table 2).

Bivariate analysis showed that male smokers were twice as likely to make quitting attempts compared to female smokers (OR = 2.07, 95% CI = 0.57–7.45). Smokers who are willing to quit smoking in the future were more likely to have made quitting attempts (OR = 1.36, 95% CI = 0.40–4.45) (Table 3).

## 4. Discussion

Among the current tobacco smokers in this study, 66.5% of the students had made an attempt to quit smoking in the past. Similar results were observed in GYTS Nepal 2000 and GYTS Nepal 2007, where the proportion of students who made quit attempt was 77.7% and 93%, respectively [8, 9]. In another study from Pokhara, Nepal, 63.3% of the currently smoking adolescents had actually tried to quit in the past [10]. In GYTS India-Tamil Nadu (2000), the proportion of students who had tried unsuccessfully to stop smoking was 73% [11]. Analysis of quitting behaviors among US high school students has shown that 72.9% of ever-daily smokers had tried to quit smoking [1]. It has also been mentioned that, among high school students who are current smokers, 50% have tried to quit smoking cigarettes during the 12 months before the survey [12].

Adolescent smokers often want to stop smoking and frequently attempt smoking cessation but are unable to maintain long term abstinence mainly because, just like adults, they are dependent on tobacco and experience withdrawal symptoms. The continuum of smoking behavior among children has been described in stages of preparation, trying, experimentation, regular smoking, and nicotine dependence. Although the persons who have smoked can discontinue at any stage, the attempt to quit becomes more difficult as smokers progress through the continuum and become increasingly dependent on nicotine. It is known that there are multiple nicotinic receptors in the brain that are stimulated upon intake of nicotine and there is higher level of brain nicotinic receptors in smokers compared to nonsmokers [13]. Despite lower levels of consumption, adolescent daily smokers experience nicotine dependence and the majority report experiencing withdrawal symptoms. The most commonly reported withdrawal symptom is craving or a strong desire to smoke [4]. Difficulty dealing with stress and feeling of anger have also been identified as common withdrawal symptoms and reasons for relapse [4]. In a study by Myers and Kelly in 2006, majority (63%) of adolescents had previously attempted to quit smoking and 70% had returned to smoking within a month of quitting [14]. In our study, those who attempted to quit could do so only for an average of five months, which highlights the fact that the chances of relapse after quitting are always high, more so in the adolescents.

Almost one-third of the current smokers in our study believed that smoking is harmful to their health because of which they attempted to quit. Nearly 10% of the current smokers actually experienced detrimental health effects of smoking like chest pain and cough. A study conducted among 146 smoking and ex-smoking patients, aged 14 years and above, showed that more than half (63%) of the smokers had made quitting attempts due to health reasons [15]. Symptoms like shortness, coughing spells, phlegm production, wheezing, and diminished overall health have been reported among adolescent smokers compared to their nonsmoking counterparts [16]. More emphasis needs to be put on the health benefits of quitting smoking to decrease the chances of relapse after quitting.

Nearly 60% of adolescent students who were unwilling to quit smoking in the future expressed the reason that it is already a habit. In a similar internet based study, most smokers attributed inability to stop smoking to addiction (88%), habit (88%), and stress (62%). Smokers often develop intentions to stop smoking on the basis of beliefs about the advantages and disadvantages, perception of others' views that they care about, and their perception of likelihood to success [15]. Unwillingness is a hindrance to quitting and such adolescents need to be motivated to quit smoking and relapse-prevention interventions should be employed. A softer and gradual approach should be considered for smokers unable or not interested in giving up rather than giving up abruptly [17].

Countries which have implemented antitobacco laws have strongly favored policies that prevent tobacco use among young people. Nepal has taken a large step in its campaign against tobacco by increasing the surface area of all tobacco packaging with graphic warning against tobacco from 75% to 90%. Australia has been implementing plain packaging legislation since 2012. A cross-sectional tracking survey of cigarette smokers revealed that plain packaging was associated with increased thinking about quitting and quit attempts [18]. Taking this evidence into serious consideration, health authorities of Nepal should take positive steps to amend the policy towards plain packaging in order to protect the youth from the harms from cigarette smoking and other tobacco products.

A strong curriculum to educate students about the detrimental effects of smoking on health is warranted. There should be regular school mental health program to motivate the adolescents who are actively smoking and help them quit smoking. Professional help should be provided to those adolescents who want to quit to increase the chances of remaining abstinent for prolonged time.

There were few limitations in our study. The data of this study apply only to the school going youth and are not representative of all the persons of this age group. Various factors such as being advised to quit by a health care provider, cost of cigarette, and awareness of harms of tobacco have been found to influence quitting attempts [19]. Such variables were not studied in depth in this study. Our study only presents a cross section of quitting attempts owing to the study design. Longitudinal studies are recommended to study the transition of current smokers through various stages of smoking.

## 5. Conclusion

Nicotine is one of the most difficult substances to quit owing to its strong biological basis of dependence. Despite the fact that the majority of adolescents attempted to quit smoking, the relapse rate is high. More alarming is that only a third of the adolescents who attempted to quit expressed that smoking is injurious to health. This demands attention to emphasize on educating the students about the harmful effects of smoking. Detrimental health effects of smoking like chest pain and cough can be used as a motivating factor to quitting. This can be especially important to help current smokers who are planning to quit, as a large majority of them are willing to quit smoking sometime in future.

## Figures and Tables

**Table 1:** Perceived reasons behind attempt to quit smoking (*n* = 121).

Reason	Frequency	Percentage
Believed smoking is injurious to health	43	35.5
Parents' instruction to quit	26	21.5
Did not want to continue smoking	13	10.7
Chest pain/cough	11	9.1
No specific reason	9	7.4
Friend's advice to quit	8	6.6
To test oneself	4	3.3
To be fit to compete in sports	2	1.7
Lack of money	2	1.7
Other reasons	3	2.5

**Table 2:** Perceived reasons behind unwillingness to quit smoking (*n* = 15).

Reason	Frequency	Percentage
Smoking is a habit	9	60
Cannot succeed to give up smoking	3	20
Cannot live without smoking	2	13.3
I like smoking	1	0.5

**Table 3:** Factors associated with quitting attempts (*n* = 182).

Characteristics	Quit attempt		OR (95% CI)	*p* value
	No	Yes		
Gender				
Female	5 (50)	5 (50)	1	0.307
Male	56 (32.6)	116 (67.4)	2.07 (0.57–7.45)	

Family type				
Joint	26 (37.7)	43 (62.3)	1	0.352
Nuclear	35 (31)	78 (69)	1.34 (0.71–2.52)	

Caste				
Brahmin/ chhetri	6 (17.6)	28 (82.4)	1	0.079
Janajati	48 (36.4)	84 (63.6)	1.63 (0.75–3.58)	
Dalit/terai major caste	7 (43.8)	9 (56.3)	1.61 (0.51–5.06)	

Type of school				
Government	6 (33.3)	12 (66.7)	1	0.986
Private	55 (33.5)	109 (66.5)	0.99 (0.35–2.78)	

Parental tobacco use				
Absent	37 (35.2)	68 (64.8)	1	0.566
Present	24 (31.2)	53 (68.8)	1.2 (0.64–2.24)	

Willing to quit				
No	6 (40)	9 (60)	1	0.579
Yes	55 (32.9)	112 (67.1)	1.36 (0.40–4.45)	

Heard of antitobacco law				
No	49 (34)	95 (66)	1	0.776
Yes	12 (31.6)	26 (68.4)	1.11 (0.52–2.40)	
